# Account of a Case in Which Sixteen Sewing Needles and Four Pins Were Extracted from the Wrist

**Published:** 1845-03

**Authors:** 


*Account of a Case in which sixteen sewing needles and four pins, were extracted from the Wrist.*—In compliance with your request, I submit to you the following statement of the very interesting case of which I spoke to you some days since.

About the 21st or 22d of September last, I was called to see a young lady,

Miss P——, with her hand and wrist much swollen and inflamed, and stiff, in consequence, as she supposed, of a sprain. The pain was of the acute lancinating kind, and was much increased by the least attempt to move or bend the wrist. She was ordered to keep it perfectly at rest and apply evaporating lotions.

Sunday, Sept. 29th, called again, and found the hand and wrist of nearly normal appearance, but she was not able to bend the joint without violent pain, extending up the whole length of the arm. On examination, I discovered on the back of the wrist, a short distance below the extremities of the radius and ulna, and near the middle of the wrist, just under the skin, some foreign body, apparently confined between the bones. Supposing from her account that it must be a small spiculum of bone, I made an incision directly over the body, and having by a probe ascertained its exact position, I introduced a small pair of forceps, and to my great surprise, extracted three pieces of needles. During the ensuing week, at different times I extracted other pieces, in all, eleven whole needles and four pins without heads. Since that time, my father, Dr. Gordon P. Spencer, has extracted and sent to me two others. In all sixteen needles and four pins have been extracted—three of the needles were broken into numerous pieces, all were much corroded, and most of them considerably bent. How these bodies came there, it is impossible to say, but from the manner in which they were confined, the depth from which they were taken, and the whole appearance of the parts as well as of the needles, it seems impossible that she could have voluntarily introduced any one that was extracted. Some were found as much as two inches above the incision, laying directly between the bones. In conclusion, I would state, that nearly all of these pins and needles were removed in the presence of some of our most respectable citizens, and they are all now deposited in the Museum of the Jefferson Medical College of Philadelphia.

H. GORDON P. SPENCER.

*Philadelphia, Feb.* 12, 1845.

Notwithstanding the difficulties mentioned by our young correspondent, without intending to impeach the character of the young lady for morality, we are nevertheless of opinion that she did voluntarily introduce all the pins and needles that were taken from her wrist. How else could they get there? Strange aberrations of mind occasionally occur, especially in hysterical females, under the influence of which they are guilty of acts greatly at variance with their general character.

In the *London Medical Intelligencer of Nov.* 1822, we have a notice of a case published in the Medical and Physical Journal by Dr. C. Otto, of Copenhagen, of "a dreadful case of hysteria, which for several years, appeared under the most varied and violent forms, such as stupor, delirium, dreadful cramps, tetanus, fevers, vomiting of blood, &c. At last, a tumor was perceived on the abdomen, which was opened, and a common needle was extracted. A second appeared in the loins, and another needle was found; in short, between Feb. 1819, and August, 1820, *two hundred and seventy three needles were extracted:* 39 of them being from the left lumbar region, 22 from the left breast, 41 from the epigastrium, &c. Whenever a needle was near the skin, the most acute pain, fever, hiccough, and vomiting of blood, were excited: in the intervals, however, the girl was quiet; indeed, during the fifteen years, which this case has occupied, the patient has been occasionally in perfect health, and no needles have been extracted for more than two years." *The Intelligencer* mentions another case in which "the foolish creature cajoled a number of medical men, and was very successful in extracting money from them, by permitting them to extract needles from various parts of her body."—*Med. Examiner.*

